# First records of the pest leaf beetle *Chrysolina* (*Chrysolinopsis*) *americana* (Linnaeus, 1758) (Coleoptera, Chrysomelidae) in Cyprus - a study initiated from social media

**DOI:** 10.3897/BDJ.9.e61349

**Published:** 2021-02-12

**Authors:** Michael Hadjiconstantis, Christos Zoumides

**Affiliations:** 1 Association for the Protection of Natural Heritage and Biodiversity of Cyprus, Nicosia, Cyprus Association for the Protection of Natural Heritage and Biodiversity of Cyprus Nicosia Cyprus; 2 Energy, Environment & Water Research Center, The Cyprus Institute, Nicosia, Cyprus Energy, Environment & Water Research Center, The Cyprus Institute Nicosia Cyprus

**Keywords:** Rosemary beetle, distribution, pest, host plant, citizen science.

## Abstract

The leaf beetle Chrysolina (Chrysolinopsis) americana (Linnaeus, 1758), commonly known as the Rosemary beetle, is native to some parts of the Mediterranean region. In the last few decades, it has expanded its distribution to new regions in the North and Eastern Mediterranean basin. *Chrysolina americana* feeds on plants of the Lamiaceae family, such as *Rosmarinus officinalis*, *Lavandula* spp., *Salvia* spp., *Thymus* spp. and others. *Chrysolina americana* is considered a pest, as many of its host plants are of commercial importance and are often used as ornamentals in house gardens and green public spaces. In this work, we report the first occurrence of *C. americana* in Cyprus and we present its establishment, expansion and distribution across the Island, through recordings for the period 2015 – 2020. The study was initiated from a post on a Facebook group, where the species was noticed in Cyprus for the first time, indicating that social media and citizen science can be particularly helpful in biodiversity research.

## Introduction

There are currently 11 known *Chrysolina* species on the Island of Cyprus (Bieńkowski 2001, Bieńkowski 2010, Gruev 1995). In this study, we confirm the presence of one more species, Chrysolina (Chrysolinopsis) americana (Linnaeus, 1758). Despite its name, this species has not yet been found in the Americas. In the original description (Linnaeus 1758, page 372), Linnaeus wrote “Habitat in America”. It seems that he mistakenly assumed the specimen had been collected in the Americas. The native range of the Rosemary beetle includes Mediterranean countries, such as Albania, Croatia, France, Greece, Italy, Malta, Portugal, Slovenia, Spain, Serbia, Macedonia, Algeria, Morocco, Tunisia and Turkey (Beenen and Roques 2010, Kippenberg 2010, Pasqual C et al. 2017). From 1936 onwards, the species expanded its native range and became invasive in many northern European countries (e.g. Belgium, United Kingdom, Austria, Netherlands, Latvia and Germany), as well as in Israel (Bieńkowski and Orlova-Bienkowskaja 2018, Friedman 2016).

The adults and larvae of *C. americana* feed on the leaves of plants in the family Lamiaceae, such as *Rosmarinus officinalis, Lavandula* spp., *Salvia* spp., *Thymus* spp. and others (Friedman 2016). Some of these host plants are popular aromatic plants and are commonly used in house gardens and in green public spaces (e.g. in urban parks and on roadsides). They are also cultivated commercially and have multiple usages, such as in cooking and as cosmetic aromatic ingredients. As *C. americana* can cause damage to the foliage of these plants, it is considered a pest of such crops. Plants like *Rosmarinus officinalis* and *Lavandula* spp. are often traded for these uses between many countries. This fact is helping the expansion of the distribution of *C. americana*, which was probably introduced in some countries along with the imported plants (Bieńkowski and Orlova-Bienkowskaja 2018). Adults and larvae of the Rosemary beetle can cause a small amount of damage to the leaves of host plants (MacLeod 2002). In cases where the beetle is abundant in commercial plantations of these plants, it can cause negative economic consequences as it damages usable leaves, thereby reducing the harvested yields (Bieńkowski and Orlova-Bienkowskaja 2018).

The views on the flight capability of the Rosemary beetle are conflicting. MacLeod (2002) mentioned that *C. americana* is flightless and spreads slowly. On the other hand, Beenen and Roques (2010) reported that this species has good flight capacity and can disperse naturally over long distances.

The Rosemary beetle was not reported in Cyprus until 2015 when a lady spotted the species. She found a large number of Rosemary beetles on a *Rosmarinus officinalis* in her garden and she posted a photo in the Facebook group ‘Biodiversity of Cyprus', requesting identification of the species. The authors identified the species and communicated with her to collect specimens for this first record.

The objectives of this work are (i) to report the presence and confirm the reproduction of *C. americana* on the Island of Cyprus, (ii) to record its distribution and use of host plants over the past 5-year period (2015–2020) and (iii) to test, though experimentation, the flying ability of the beetle.

## Material and methods

### Reproduction and current distribution

From 2015 to 2020, multiple excursions were undertaken across the Island of Cyprus to locate *C. americana*. In particular, we searched for larvae and mating adults, in order to confirm the presence and local breeding of the species. We also searched repeatedly and on an annual basis certain localities with a strong presence of the host plants, grown both in natural and cultivated (e.g. garden) conditions. In addition, we checked new localities to record the dispersal of the species at new sites. The excursions were undertaken throughout the 5-year period. Road edges with planted *Rosmarinus officinalis* were frequently checked, as well as every other locality where we noticed the presence of the host plants. Rosemary is a common, native and widely-grown species in Cyprus and is planted at road edges, in house gardens and in parks. Additionally, several previously-recorded host plant species are native to Cyprus and occur naturally in the environment; such localities were also examined.

In addition to site visits, we frequently checked for records of *C. americana* in online social media groups on the fauna of Cyprus (e.g. in Facebook) where photos of insects are posted for identification. These resources were examined at least once per month.

### Flying ability experiment

To confirm the flying ability of *C. americana*, a small tree branch was attached on a piece of modelling clay in the middle of a metal bowl with 2 cm of water (Figure 2J). We placed 10 adults of *C. americana* on the branch and the bowl was placed on a sunny outdoor place for 24 hours. This process was repeated four times in warm, outdoor conditions and the activity of the beetles was checked every 1 hour. During the third and fourth trial, the experiment was carried out by adding a Rosemary plant at 1 m distance from the bowl, to check if that triggers the beetles to fly. It should be noted that different individuals were used each time the experiment was carried out. Individuals used for the experiment were collected from Aglantzia and Strovolos, in Nicosia District. The dates the experiment was performed are the following: 8 Feb 2020, 10 May 2020, 12 Aug 2020 and 5 Oct 2020. We have also attempted to provoke and encourage individuals to fly in field conditions, at the localities that were found, by moving the beetles on non-host plants and rocks near host plants.

Additionally, we examined the wings of more than 20 specimens of both sexes to confirm if the species is brachypterous (i.e. if it has reduced wings).

## Results

### Occurrence, reproduction, current distribution

**Figure 1.**
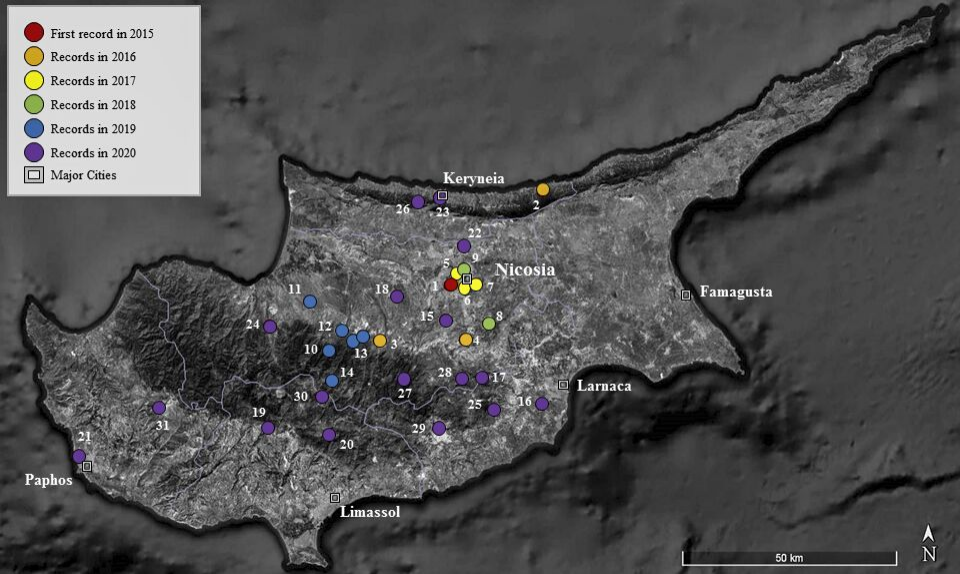
Locations of *Chrysolina americana* records, with numbers indicating the records in Table 1 and colours indicating the chronological order in the legend since 2015; the first record at Strovolos is indicated with a red dot.

**Figure 2.**
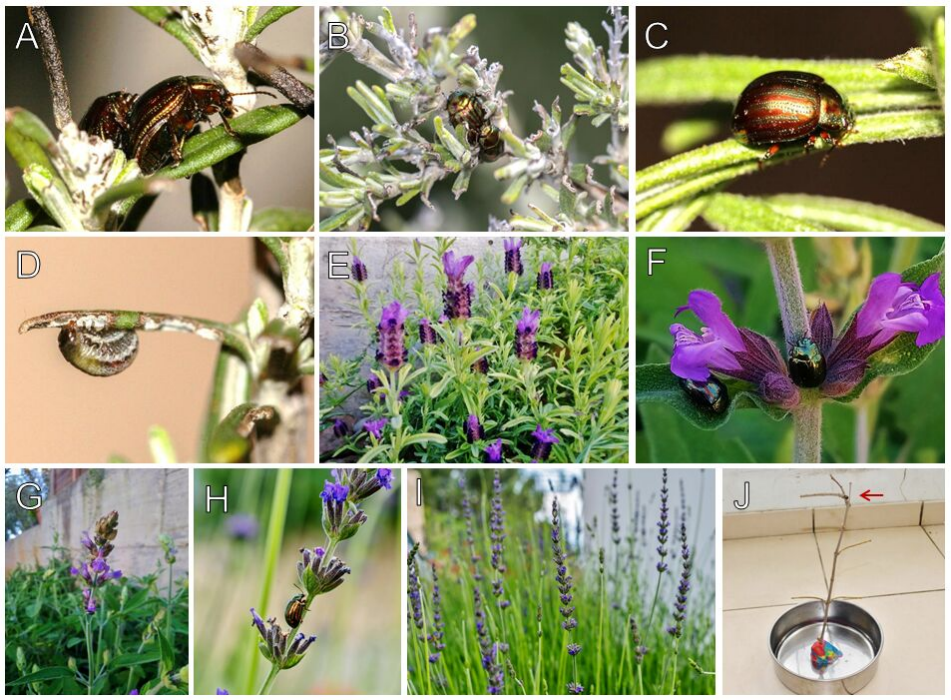
**A** – **C**: adults of *Chrysolina americana* on Rosmarinus officinalis; **D**: larva; **E**: hosted *Lavandula stoechas*; **F** – **G**: hosted *Salvia officinalis*; **H** – **I**: hosted *Lavandula angustifolia*; **J**: flying ability experiment set up with red arrow showing the remaining individual.

The species *Chrysolina americana* was recorded at 31 localities in Cyprus, as shown on the map (Fig. 1). The records occurred in five host plants: *Rosmarinus officinalis* L., Lavandula stoechas L., *Lavandula angustifolia* Mill., *Salvia fruticosa* Mill. and *Salvia officinalis* L. (Fig. 2). From the recorded host plants, *Rosmarinus officinalis*, *Lavandula stoechas* and *Salvia fruticosa* are native to Cyprus. Additionally, *Lavandula angustifolia* is a naturalised species and grows naturally (Tsintidis et al. 2002). The host plants identification was performed by the authors, using the books of Christofides 2017 and Tsintidis et al. 2002. In controversial cases, a flora specialist was asked to advise and confirm the validity of the identification. Both larvae and adults were present simultaneously at the majority of the recorded localities. In many cases, adults were mating (Fig. 2A and B). The localities, dates, coordinates, host plants and altitudes of the records are shown in Table 1. Records of the same areas are shown on Fig. 1 and have been merged in Table 1.

Regarding reports in social media, we found 12 cases where photos of the species had been posted in the Facebook group ‘Biodiversity of Cyprus’. Some of these records were at the same or a nearby locality to our previous records. When needed, we contacted the people posting the records to ask about the location and the date in which the photos were taken. In the majority of these cases, we visited the recording area for confirmation.

### Flying ability experiment of Chrysolina americana

Regarding the flying ability experiment, in all four replicates, some of the beetles fell in the water and were repositioned to the branch. No individual beetle was found outside the bowl or attempted to fly. On the third replicate, we left the beetles on the branch for 9 days. Most individuals (9 out of 10) eventually drowned in the water during the 6^th^ day. The remaining individual stood on the branch for 9 days; see the red arrow in Fig. 2J. On the fourth replicate, three beetles remained alive on the branch for 15 days.

In the case of the field experiment, beetles were placed on high branches of bushes and we observed their behaviour for 1 hour. None of the beetles attempted to fly. In all cases, the beetles walked randomly on the branches or fell off when they felt threatened. Similarly, beetles placed on rocks near the host plants walked randomly, but none of them attempted to fly. We also noticed that the beetles opened their wings only in some cases, i.e. when they fell upside down and in the attempt to reposition themselves.

**Figure 3.**
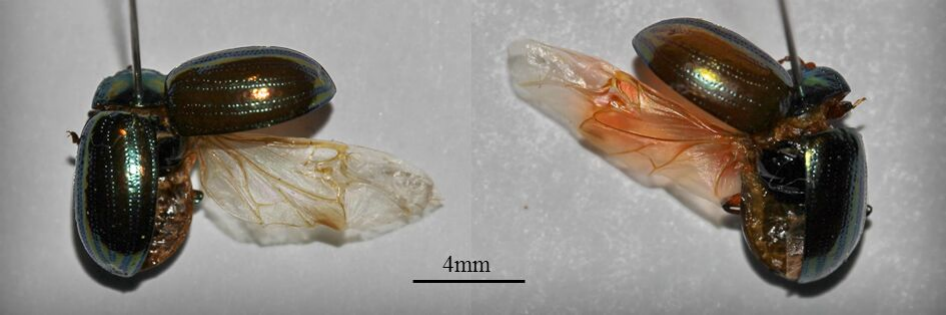
Two specimens of *Chrysolina americana* showing a wing. Photographs were taken with the same zoom. The camera was a Canon EOS 600D with a Canon EF 100 mm f/2.8L Macro IS USM lens. Scale bar of 4 mm is shown.

From the examination of the beetles’ wings, the species seems to have normal length wings for flight and it is not brachypterous. Two specimens showing the wing are presented in Fig. 3.

## Discussion

This paper confirms the presence and reproduction of *C. americana* in Cyprus. Since its first appearance in March 2015, the species has been recorded in many areas and it has spread across most of the Island. In particular, at the time of writing (November 2020), it has been recorded at almost all districts of Cyprus, except Famagusta. Based on a 5-year period of records, the altitudinal range of the beetle varies between 34 m a.s.l. at the coastal area of Dromolaxia (March 2020) to 1037 m a.s.l. at the mountainous area of Agros (May 2020), indicating its plasticity to various climatic conditions and environments. The main host plant of the species in Cyprus is the Rosemary, especially on roadside plantations.

Both the indoors and outdoors flying experiments cannot confirm that *C. americana* is able to fly, despite the wings' examination showing that the species is not brachypterous (Fig. 3); this finding is not in agreement with MacLeod (2002). Nevertheless, *C. americana* could be capable of flight. A definitive answer to the question of whether it can fly or not can be given in a future examination of its flight muscles. From previous studies on other leaf beetles, such as *Galeruca tanaceti* (Linnaeus, 1758) (Beenen 2005), it seemed that a part of the fresh population of specimens had well-developed flight muscles and the old (post-summer diapause) specimens did not. Beenen 2005) findings suggest that, although we used individual specimens from different seasonal periods across a year for our experiments, not all populations may include specimens capable of flight. Therefore, it remains to be determined in a future study whether this species can fly or not.

Although we are not sure of the flying capabilities of the Rosemary beetle, it has evidently a very good dispersal ability as it has spread over almost all of Cyprus in a period of five years from the first record. We are certain that the beetle was not introduced to Cyprus with the Rosemary plant of the first recording because the particular plantation existed years before the beetle appeared and the diameter of the Rosemary trunk at the time of recording was larger than 4 cm, indicating a relatively old Rosemary plant. We do not know if our first record at Strovolos (March 2015) coincides with the original year of introduction of the species to the Island or whether it arrived at an earlier date. What we can confirm, however, is that the species was recorded 95 km away (in Chloraka) from the first recorded site, 5 years later (April 2020, see Table 1). Such fast dispersal ability is unexpected for a flightless species. Some possible explanations include the frequent transfer of host plants by people, which potentially helps spread the species on the Island of Cyprus. Considering that the Rosemary beetle cannot reach Cyprus by flight, we assume that it was imported with its host plants relatively recently. This assumption is supported by new records in many countries outside its natural distribution (Bieńkowski and Orlova-Bienkowskaja 2018).

It is interesting to note that *C. americana* has also recently been reported from Israel with the presumed first record being December 2014 (Friedman 2016), a few months before our first record. This species probably arrived in Israel from imported host plants as it was first found close to the main port of Haifa (Friedman 2016). However, it is not unreasonable to assume that *C. americana* may have dispersed naturally into Israel. That is, from south-western Turkey, where it occurs naturally (Bieńkowski and Orlova-Bienkowskaja 2018), eastwards along the Mediterranean coast of Turkey to Syria, Lebanon and into Israel; Haifa is approx. 30 km south of the border with Lebanon. This hypothesis can be confirmed if further research identifies *C. americana* in Syria and Lebanon and if its importation seems unlikely. However, if this is found to be the case, then it seems likely that *C. americana* may have been accidentally imported to Cyprus from southern Turkey into the northern part of Cyprus. The fact that the presumed first record was in Nicosia, with the closest ports being in Keryneia (21 km away), lends weight to this hypothesis.

## Conclusions

Although the species has not been part of Cyprus fauna until now, it is a Mediterranean species that seems to be well adapted to the climatic conditions of Cyprus. In addition, owing to the fact that (i) a large number of its host plants are native to Cyprus or naturalised and are found growing naturally or in cultivated conditions across the Island (Tsintidis et al. 2002) and (ii) the species (both larvae and adults) have been found at forest areas, we can safely conclude that it is established. From our observations, the species can cause minor damage on the foliage of its host plants. Significant damage to the host plants has been reported only on a few urban locations and, in particular, at house gardens; these are cases where *C. americana* is abundant and has completely damaged the plant’s foliage. This behaviour could be linked to areas with well-maintained gardens, for example, in the absence of natural predators, the beetle can thrive and can potentially cause significant damage. At the moment, no such extreme case has been reported in commercial cultivations or protected areas, yet this behaviour of the species may cause significant problems to farmers in the near future. As *C. americana* is a Mediterranean species, its natural enemies are also part of the fauna of Cyprus. Known natural enemies are *Anaphes chrysomelae* (Bakkendorf) (Hymenoptera: Mymaridae) which parasitised the eggs of *C. americana* in Italy (Tarla and Tarla 2017) and parasitic flies (Diptera: Tachinidae) which parasitised the larvae, such as *Meigenia dorsalis* (Meigen, 1824), *Meigenia mutabilis* (Fallén, 1810), *Macquartia dispar* (Fallén, 1820), *Macquartia tenebricosa* (Meigen, 1824) and *Macquartia tessellum* (Meigen, 1824) (Cerretti and Tschorsnig 2010). From those enemies, *Macquartia tessellum* (Meigen, 1824) is present in Cyprus (Bergstrom et al. 2017). Keeping in mind that the commercially-grown aromatic plants are organically cultivated and are typically used in cooking, farmers can use pesticides as a last resort; alternatively, farmers can prevent damage by maintaining the appropriate conditions for the natural enemies of the species. Additionally, control of the beetle population can be achieved by removing the beetles and larvae manually, by hand-picking, net sweeping or by shaking the plants and collecting the beetles from the ground, which, however, implies additional labour hours and cost.

As a concluding note and given that the initiation of this study was a record first reported in social media, we would like to emphasise the important role of citizen science and, in particular, the participation and contribution of people using such networking platforms in recording wildlife. Although citizen science – the collection of data relating to the natural world by members of the general public – has been a part of observations of the natural world for a very long time, digital developments in the past decade or so have vastly expanded the potential for input from citizens into the study of biodiversity and associated impacts (Bonney et al. 2014). *Chrysolina americana* is not the only recorded species in Cyprus whose presence was first known through social media. Several species were recorded in Cyprus for the first time from people posting photographs on social media and were noticed by researchers. Most of them are in the process of preparation for publication and scientists are collaborating with interested social media users in collecting more information and records for these new findings.

## Figures and Tables

**Table 1.** Records of *C. americana* from 2015 to 2020, indicating locations, District, date of collection, coordinates, host plant and altitudes.

**a/a**	**Location**	**District**	**Date of collection**	**Coordinates (lat/long)**	**Host plant**	**Altitude (m)**
1	Strovolos	Nicosia	24 Mar 2015	35.147°/33.353°	*R. officinalis*	180
2	Agios Amvrosios	Keryneia	20 Jan 2016	35.339°/33.580°	*R. officinalis*	160
3	Klirou	Nicosia	20 Mar 2016	35.031°/33.165°	*R. officinalis*	423
4	Nisou	Nicosia	25 Apr 2016	35.033°/33.381°	*R. officinalis*	269
5	Eleutherias square	Nicosia	4 Apr 2017	35.168°/33.361°	*R. officinalis*	152
6	The Cyprus Institute	Nicosia	9 Apr 2017	35.141°/33.381°	*R. officinalis*	174
7	Aglantzia, Uchall	Nicosia	13 Apr 2017	35.147°/33.409°	*S. officinalis*	141
8	Agios Sozomenos	Nicosia	9 Mar 2018	35.066°/33.440°	*R. officinalis*	179
9	Frederick University	Nicosia	26 Apr 2018	35.180°/33.380°	*R. officinalis*	138
10	Xyliatos Dam Picnic Sit	Nicosia	7 Apr 2019	35.010°/33.037°	*R. officinalis*	530
11	Kato Koutrafas	Nicosia	14 Apr 2019	35.112°/32.988°	*R. officinalis*	207
12	Agia Marina (Xyliatou)	Nicosia	11 Mar 2019	35.051°/33.070°	*R. officinalis*	390
13	Mitsero	Nicosia	11 Mar 2019	35.040°/33.123°	*R. officinalis*	400
14	Platanistasa	Nicosia	21 Dec 2019	34.948°/33.043°	*R. officinalis*	940
15	Tseri	Nicosia	11 Feb 2020	35.074°/33.332°	*R. officinalis*	282
16	Dromolaxia	Larnaca	14 Mar 2020	34.899°/33.574°	*R. officinalis*	34
17	Mosfiloti	Larnaca	20 Mar 2020	34.954°/33.425°	*R. officinalis*	250
18	Ayioi Trimithias	Nicosia	3 Apr 2020	35.123°/33.209°	*L. stoechas*	258
19	Kouka	Limassol	5 Apr 2020	34.851°/32.887°	*R. officinalis*	737
20	Louvaras	Limassol	5 Apr 2020	34.837°/33.039°	*R. officinalis*	725
21	Chloraka	Paphos	7 Apr 2020	34.791°/32.407°	*R. officinalis*	75
22	Mandres	Nicosia	15 Apr 2020	35.226°/33.378°	*R. officinalis*	155
23	Keryneia Town	Keryneia	19 Apr 2020	35.327°/33.317°	*R. officinalis*	62
24	Flasou	Nicosia	21 Apr 2020	35.062°/32.888°	*R. officinalis*	320
25	Stavrovouni Forest	Larnaca	23 Apr 2020	34.888°/33.454°	*S. fruticosa*	230
26	Karmi	Keryneia	4 May 2020	35.318°/33.264°	*L. angustifolia*	280
27	Mandra tou Kampiou	Nicosia	11 May 2020	34.952°/33.228°	*L. stoechas*	652
28	Mathiatis	Nicosia	16 May 2020	34.951°/33.373°	*L. stoechas*	354
29	Kato Drys	Larnaca	23 May 2020	34.851°/33.316°	*R. officinalis*	544
30	Agros	Limassol	27 May 2020	34.918°/33.021°	*R. officinalis*	1037
31	Statos	Paphos	1 June 2020	34.890°/32.608°	*R. officinalis*	793
